# Family Support, Multidimensional Health, and Living Satisfaction among the Elderly: A Case from Shaanxi Province, China

**DOI:** 10.3390/ijerph17228434

**Published:** 2020-11-14

**Authors:** Lijian Wang, Liu Yang, Xiaodong Di, Xiuliang Dai

**Affiliations:** School of Public Policy and Administration, Xi’an Jiaotong University, Xi’an 710049, China; wanglijian2@mail.xjtu.edu.cn (L.W.); dxd6794860@stu.xjtu.edu.cn (X.D.); xiuliangdai@stu.xjtu.edu.cn (X.D.)

**Keywords:** elderly individuals, family support, multidimensional health, living satisfaction, China

## Abstract

The current study investigated the association between three types of family support and living satisfaction of elderly individuals in China, and paid particular attention to the possible mediating role of the elderly population’s multidimensional health. A cross-sectional survey was conducted in 2019, and 938 elderly people from seven counties (districts) of China’s Shaanxi province were enrolled. Multivariable linear regression and mediation effect analysis were employed to examine the integrated relationships among these variables. The results showed that emotional support and decisional support from families were positively related to the living satisfaction of elderly individuals (β = 0.101, *p* = 0.000; β = 0.263, *p* = 0.000), while the relationship between daily living support and living satisfaction was not significant (β = 0.017, *p* > 0.05). The mediation examination further demonstrated that both mental state and social integration mediated the association between emotional support and living satisfaction, as well as the association between decisional support and living satisfaction, but a mediating effect of physical health was not observed. These results indicate the pathways in the relationships of different types of family support to living satisfaction via mental state and social integration, having significant implications for enhancing the living satisfaction the elderly.

## 1. Introduction

With the increased average expected lifespan and decreased fertility rate, China is experiencing rapid population aging, and is becoming the country with the largest elderly population in the world. By the end of 2019, the number of people aged over 65 in China reached over 175.99 million, accounting for 12.57% of the country’s total population [1]. It is predicted that the number of people aged over 65 in China will increase to approximately 400 million by 2050, or 26.9% of the country’s population [2]. Living satisfaction, defined as an individuals’ global assessment of their important life domains, has been identified as one of the most important criteria for successful aging [3,4,5]. Therefore, it is crucial to investigate the critical determinants of living satisfaction among older populations in China.

As with other East Asian countries, in China, an individual’s perception of family relationships is mainly influenced by the filial piety, a central concept in Confucian culture [6]. Thus, family members, especially adult children, are expected to take care of and support the elderly, and the family support seems to become an important source of living satisfaction and well-being for aging adults [7,8]. However, due to the decline in fertility and the development of urbanization and industrialization, the traditional intergenerational co-residence mode has been decreasing and the traditional perception has been eroding in the past two decades [9,10,11]. As such, it is unclear whether elderly individuals’ living satisfaction is still bonded to family support in the current Chinese society.

Furthermore, growing evidence suggests that living satisfaction is closely associated with older adults’ physical health, mental health, and social well-being [12,13]. Thus, this study first investigated the impact that family support has on elderly individuals’ living satisfaction. On this basis, we paid particular attention to the possible mediating effect of health and further explored the mediational pathways in the relationship between different types of family support and living satisfaction via multidimensional health.

## 2. Literature Review and Conceptual Framework

### 2.1. Family Support and Living Satisfaction

As people get older, their social network tends to shrink and family support, as a vital component of elderly people’s social support, has been found to have greater importance than non-family support for elderly people [14,15]. A large body of research has demonstrated a positive association between family support and elderly people’s living satisfaction. Grundy and Henretta (2006) found that support stemming from within the family system may be of particular importance to well-being in older adults [7]. Cheng and Chan (2006) suggested that support from family members is beneficial for the well-being of older groups [16]. Zulfitri, Sabrian, and Herlina (2019) pointed out that family social capital, including family interactions, family relationships, family support, and family structures, has a significant impact on well-being at an older age in East Asian countries and communities [17].

Existing studies often take family support as a single variable, while family support is a complex construct with multiple aspects [18]. Growing research has reported that different dimensions of family support may have differing associations with elderly individuals’ living satisfaction. Zunzunegui, Beland, and Otero (2001) reported that emotional and instrumental support may have differing associations with well-being [19]. Merz and Consedine (2009) suggested that emotional support is generally associated with higher well-being, whereas instrumental support is related to decreased well-being [8]. Chen and Jordan (2016) divided family support into financial support and instrumental support, and only observed the primary importance of receiving financial support to enhance elderly living satisfaction [20]. However, inconsistent conclusions have been drawn in some studies in the context of China. Chinese people view family support as an important manifestation of filial piety, and lack of family care results in the elderly being psychologically disappointed and endangers their security in old age, leading to a reduction in life satisfaction [21]. Peng et al. (2019) revealed that elderly parents are less satisfied with their lives when they have no flow of exchange in daily living support, and are more satisfied when they are under-benefited in terms of financial support and over-benefited or reciprocal in emotional support [22].

According to the support/efficacy theory, social relationships, such as family relations, affect well-being by instilling in an individual a sense of self-efficacy and self-esteem [23]. The underlying implication is that there might be a positive relationship between the esteem received from family members and elderly people’s life satisfaction. Therefore, in consideration of the fact that elderly individuals are most likely to heighten their self-esteem when they have no doubt of their own decision-making abilities [24], we took decisional support to measure elderly people’s esteem received from their families and further explored whether it is related with the elderly people’s satisfaction with life.

On this basis, we examined three dimensions of family support, namely, daily living support, emotional support, and decisional support, as well as their impact on elderly individuals’ living satisfaction in the context of China. Given the findings of previous studies and the culture difference between Chinese society and Western societies (the filial piety culture), we first developed the following research hypotheses:

**Hypothesis** **1.** *Receiving daily living support from families has a positive impact on elderly individuals’ living satisfaction.*

**Hypothesis** **2.** *Receiving emotional support from families has a positive impact on elderly individuals’ living satisfaction.*

**Hypothesis** **3.** *Receiving decisional support from families has a positive impact on elderly individuals’ living satisfaction.*

### 2.2. Multidimensional Health and Living Satisfaction

Elderly people’s living satisfaction seems to be associated with numerous factors, among which health status stands out as an important one [25]. Some studies have suggested the significant association between physical health, mental health, and living satisfaction. Pinto, Fontaine, and Neri (2016) found a negative relationship between number of chronic conditions and living satisfaction [26]. Pinto (2013), Celik (2017), Lopez-Ortega (2017), and Ng (2017) reported that the variables that are negatively related to living satisfaction in older adults included a lower self-reported health status, a decline in physical health, and depressive symptoms [27,28,29,30]. Camacho (2019) suggested that self-rated health, affect, interpersonal activities, and perceived safety in the street are significantly associated with high life satisfaction [31].

As per the World Health Organization (WHO), health is a state of physical, psychological, and social well-being, not just the absence of disease or weakness [32]. However, few studies have taken social well-being as a health variable to explore its impact on the living satisfaction of the elderly. Social health is a complex concept and is usually explained as “well-being” or other terms rather than “health” [33]. Theoretically, social well-being of an individual contains five dimensions: Social integration, social contribution, social coherence, social actualization, and social acceptance [34]. However, in empirical studies, researchers usually measure social health by social engagement [35], social adjustment [36], social integration [31], or other variables [37]. Based on previous research, this study centered on the multidimensional health of the elderly, including physical health, mental state, and social integration, and further explored their impact on elderly individuals’ living satisfaction.

### 2.3. Family Support, Multidimensional Health, and Living Satisfaction

The findings of existing empirical studies suggest that both family support and health are considered important determinants for elderly individuals’ living satisfaction. Additionally, a variety of studies have shown that social support, including family support, has a significant impact on the health of the elderly [38]. Choi (1996) suggested that in America, social support for the elderly retards further physical and functional deterioration in older adults [39]. Lyons (2016) and Koelmel et al. (2017) demonstrated that emotional and practical support from family members can reduce the occurrence of psychological distress, thereby reducing the level of depression [40,41]. Yunli Bai (2020) suggested that communicating with children by telephone significantly improves the mental health of the rural elderly in China [42]. These results imply that family support is associated with the living satisfaction of the elderly through the mediating role of multidimensional health.

To further explain the integrated relationship among these variables, this study took three dimensions of health as the pathways through which specific family support affects elderly individuals’ living satisfaction. For daily living support, elderly individuals may benefit from their family’s basic care, which could promote their rehabilitation progress for physical problems and help them to cope with chronic diseases and multimorbidity [43]. Moreover, with daily care from their families, the elderly will have more opportunities to interact with their family members and to participate in social activities and physical exercise, reducing their sense of loneliness and the risk of mental problems and enhancing their living satisfaction. For emotional support and decisional support, encouragement, companionship, psychological comfort, and esteem from their families may encourage the elderly to increase their physical exercise and help to relieve elderly individuals’ psychological distress, as well as provide them confidence to integrate into society and to promote their social well-being. Therefore, the following hypotheses are proposed.

**Hypothesis** **4.** *Physical health mediates the association between the daily living support and living satisfaction of elderly individuals.*

**Hypothesis** **5.** *Mental state mediates the association between the daily living support and living satisfaction of elderly individuals.*

**Hypothesis** **6.** *Social integration mediates the association between the daily living support and living satisfaction of elderly individuals.*

**Hypothesis** **7.** *Physical health mediates the association between the emotional support and living satisfaction of elderly individuals.*

**Hypothesis** **8.** *Mental state mediates the association between the emotional support and living satisfaction of elderly individuals.*

**Hypothesis** **9.** *Social integration mediates the association between the emotional support and living satisfaction of elderly individuals.*

**Hypothesis** **10.** *Physical health mediates the association between the decisional support and living satisfaction of elderly individuals.*

**Hypothesis** **11.** *Mental state mediates the association between the decisional support and living satisfaction of elderly individuals.*

**Hypothesis** **12.** *Social integration mediates the association between the decisional support and living satisfaction of elderly individuals.*

**Figure 1:**
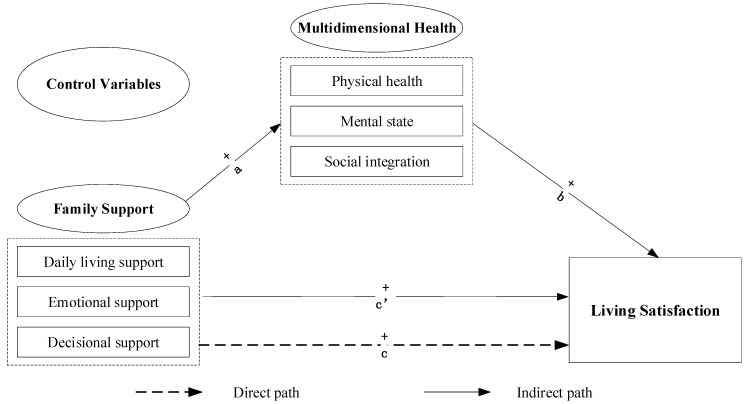
The conceptual mediation model relating to the family support, health, and life satisfaction of elderly individuals.

In summary, different types of family support and health can directly influence the elderly’s living satisfaction. Furthermore, existing studies imply that different types of family support have an impact on the multidimensional health of the elderly, which further influences their living satisfaction; that is, multidimensional health may play a mediating role between family support and the elderly’s living satisfaction. The conceptual framework of this study is presented in Figure 1.

## 3. Materials and Methods

### 3.1. Study Design and Data Collection

#### 3.1.1. Study Design and Sampling

The data used in this study were obtained through the survey used in the High-Quality Development of China’s Undertakings for The Aged organized by Xi’an Jiaotong University in 2019. A stratified sampling method was employed to yield the sample in this study. First, we selected three representative cities located in China’s Shaanxi province, namely, Baoji, Yan’an, and Hanzhong, which are characterized by rapid population aging and ranked as moderate in economic development. Second, the isometric random sampling method was used in the selection of seven counties (districts) on the basis of their per capita gross value of industrial output. Third, three or four typical urban communities and rural administrative villages were selected according to their administrative divisions. Finally, we randomly selected approximately 35 respondents among the elderly individuals in each community or village if they (1) were aged ≥60 years, (2) were able to communicate easily or communicate with the investigators’ assistance, and (3) volunteered to participate in the study. Elderly residents were excluded if they (1) could not communicate even with the assistance of the investigator (e.g., severe hearing loss, obvious language difficulties, or severe cognitive impairment) or (2) were unwilling to participate in the study.

#### 3.1.2. Data Collection

Before data collection, the investigators verbally introduced the background, content, and purpose of the study to respondents and assured them that the data involved no personal privacy information and were for research only, and verbal consent was obtained from each of them. Participation was voluntary, and the survey was anonymized. Data collection took approximately 20 min for each respondent. The survey team was composed of 6 specialists and 21 well-trained students. The information collected for this study included four parts: (1) Personal sociodemographic characteristics; (2) family support status; (3) multidimensional health status; (4) living satisfaction. The research team collected data through face-to-face interviews and surveys. During this process, investigators asked the respondents and completed the surveys based on their answers. To ensure the integrity and accuracy of the data collected, the investigators reviewed each completed survey. After eliminating surveys lacking key information, 938 valid samples were obtained, giving the survey a final response rate of 98.95%.

### 3.2. Measurements

#### 3.2.1. Dependent Variable: Living Satisfaction

Living satisfaction was measured using the Satisfaction with Life Scale (SWLS) [3]. The SWLS consists of 5 items (e.g., “Taking all things together, I am satisfied with my life”) and participants indicated how much they agreed or disagreed with each of the 5 items using a 5-point scale from 1 (very disagree) to 5 (very agree). The total score can range from 5 to 25 and a higher score indicates higher levels of living satisfaction. SWLS is one of the most widely used scales for living satisfaction, with high reliability and validity [44], and the Chinese version of SWLS has also been shown to be reliable and valid [45]. The internal consistency of the scale in this sample is high, with a Cronbach’s alpha of 0.84.

#### 3.2.2. Independent Variable: Family Support

Three types of family support were examined: Daily living support, emotional support, and decisional support. Daily living support assessed the practical daily care assistance provided by family members, especially some actual types of support such as cooking and cleaning. Daily living support was mainly reflected by the question “Can you get life care from your family/relatives when you are in need?” with response categories ranging from 1 (never) to 5 (always). The second type of family support was emotional support, which was mainly manifested in the emotional intimacy of the elderly and their family members. We assessed emotional support via the question “Do you often meet with or contact your family/relatives in one month (including telephone and mail)?” with response categories ranging from 1 (never) to 5 (always). The third indicator was decisional support, which reflected the respect and understanding provided by family members. Decisional support was mainly reflected by the question “Does your family/relatives support and respect your ideas and decisions?” with response categories ranging from 1 (never) to 5 (always).

#### 3.2.3. Mediators: Multidimensional Health

In this study, three indicators were used to measure the multidimensional health of elderly individuals: Self-rated physical health, self-rated mental state, and social integration. Self-reported health is a common and important indicator that can comprehensively and directly reflect people’s self-perception on health [46]. Therefore, in terms of the assessment regarding physical health and mental health, self-rated physical health was measured using the question “In general, how would you rate your physical health?” and self-rated mental state was assessed using the question “In general, how would you rate your mental state?” with possible answers ranging from 1 (very bad) to 5 (very good).

In terms of social integration, we assessed the social integration of elderly individuals from three dimensions: Social participation, social relationships, and social identity. Social participation was examined by asking “Do you often chat and take part in activities with your friends or neighbors in your community or institution?” with response categories ranging from 1 (never) to 5 (always). Social relationships were assessed using the question “How about the relationship with your friends or neighbors in your community or institution?” with response categories ranging from 1 (very bad) to 5 (very good). Social identity was reflected by the question “Do you feel comfortable in your community or institution?” with response categories ranging from 1 (very uncomfortable) to 5 (very comfortable). The total score can range from 3 to 15 and a higher score indicates higher levels of social integration.

#### 3.2.4. Control Variables

The data of the following sociodemographic variables were obtained because of a potential association with living satisfaction: Age, gender, marital status, household income, hukou location, education level, living mode, having any ADL (Activities of Daily Living) limitation, having any chronic disease, health insurance, and old age insurance. Age was set as a continuous variable, education and household income were set as categorical variables, and the others were set as binary variables. A description of these control variables is shown in Table 1.

### 3.3. Statistical Analysis

First, descriptive statistics (means, standard deviations, frequency distributions, and percentages) were conducted to show the characteristics of the sociodemographic variables, dependent variable, independent variables, and mediators. Second, a multivariable linear regression analysis was used to investigate the correlation between the sociodemographic variables, family support variables, multidimensional health variables, and living satisfaction. The sociodemographic variables were entered first, followed by the variables relating to family support, and then the health-related variables. Finally, to assess whether the association between family support and elderly individuals’ living satisfaction was mediated by multidimensional health, the procedure recommended by Baron and Kenny (1986) was employed first. Briefly, mediating models require (1) a significant regression path between independent variable A (different types of family support) and mediator B (three dimensions of health); (2) a significant regression path between independent variable A (different types of family support) and dependent variable C (living satisfaction); (3) a significant regression path between mediator B (three dimensions of health) and dependent variable C (living satisfaction). Full mediation occurs when the significant relationship between dependent variable A and independent variable C is no longer significant when controlling for the effect of mediator B. Partial mediation occurs when the relationship between dependent variable A and dependent variable C is reduced [47]. Then, bias-corrected and accelerated bootstrap 95% CIs (BCa CIs) were further calculated on the basis of 5000 samples, as the bootstrapping method presents the advantage of analyzing multiple mediators simultaneously [48] and minimizing the number of inferential tests, which reduces the likelihood of type-I errors [49] compared to other method. Bootstrapping is a non-parametric resampling method used for estimating direct and indirect effects in multiple mediator models. This procedure involves repeatedly extracting samples from the data by randomly sampling with replacing and estimating the indirect effect in each resampled dataset. If zero is contained within the 95% CIs, then a lack of significance is assumed [49].

## 4. Results

### 4.1. Descriptive Statistics

Table 2 summarizes the participants’ sociodemographic characteristics. Among the 938 participants, the average age was 72.19 years, with a range of 60–97 years. There were 402 (42.86%) males and 536 (57.14%) females. Of the participants, 43.28% were non-single and 56.72% were single. Participants with a household income of more than ¥100,000 per year totaled 806 (85.93%), and those with a household income of less than ¥10,000 per year accounted for 14.07% (132). Most of them were urban residents (54.80%) and lived in the home or the community (73.24%). In terms of education level, 47.65% of the participants had a low educational status, 26.33% had graduated from junior middle school, and 26.01% had a higher educational status. Only 76 (8.10%) participants reported that they had at least one ADL limitation, while 529 (56.40%) participants suffered from chronic diseases. Most of the elderly individuals had health insurance (97.44%) and old age insurance (94.56%).

Table 3 shows the living satisfaction, family support, and multidimensional heath characteristics of the participants. Overall, the elderly individuals had a mean living satisfaction score of 19.061 (SD = 3.053). In terms of family support, the mean scores of daily living support, emotional support, and decisional support were 3.472 (SD = 1.462), 4.095 (SD = 1.096), and 4.259 (SD = 0.731), respectively. The mean score of self-rated physical health was 3.383 (SD = 1.095), the mean mental state score was 4.168 (SD = 0.939), and the mean social integration score was 12.978 (SD = 2.029). Considering the different dimensions of the variables, data normalization was further conducted and the value range of each variable was 0–1.

### 4.2. Multivariate Linear Regression Analysis

Table 4 presents the correlations between the control variables, family support, multidimensional health, and the elderly individuals’ living satisfaction. In model 1, only the control variables were included. For the demographic characteristics, household income, education level, living mode, having any ADL limitation, and having any chronic disease were significantly associated with living satisfaction. Elderly individuals with a higher household income and education level reported higher living satisfaction. Those living in old age care institutions and having no ADL limitation or chronic disease were more satisfied with their life.

In model 2, the family support variables were added to examine the relationship between different types of family support and the living satisfaction of the elderly individuals. The regression results show that, when the sociodemographic variables were being controlled, both emotional support (β = 0.101, *p* = 0.000) and decisional support (β = 0.263, *p* = 0.000) had a significant and positive correlation with the elderly individuals’ living satisfaction. The estimation results also revealed that the effect of decisional support on living satisfaction was larger than that of emotional support. However, no significant association was found between daily living support and living satisfaction (β = 0.017, *p* > 0.05), which indicates that hypothesis 1 was not supported, and thus, hypothesis 4, hypothesis 5 and hypothesis 6 were not supported either.

Model 3 added the health variables and revealed that the elderly individuals with better self-rated physical health (β = 0.073, *p* = 0.000), mental state (β = 0.246, *p* = 0.000), and social integration (β = 0.199, *p* = 0.000) reported higher living satisfaction. In addition, after adding the health variables, the relationship between emotional support and living satisfaction was no longer significant (β = 0.010, *p* > 0.05). The positive and significant effect of decisional support on living satisfaction did not change, while the regression coefficient (β) decreased from 0.263 to 0.114. The results of model 3 suggest that multidimensional health might have a mediating effect on the relationship between emotional support, decisional support, and living satisfaction.

### 4.3. Mediation of Multidimensional Health between Family Support and Elderly Individuals’ Living Satisfaction

To examine the mediating effect of multidimensional health on the association between emotional support, decisional support, and living satisfaction, the mediation analysis procedure recommended by Baron and Kenny (1986) was run and the results are illustrated in Table 5. According to the three conditions of mediation analysis, first, the results of the multivariable linear regression revealed that only emotional support and decisional support were significantly associated with elderly individuals’ living satisfaction. Second, the results of the mediation analysis in Table 5 indicate that emotional support and decisional support were significantly associated with elderly individuals’ mental state (β = 0.175, *p* = 0.000; β = 0.369, *p* = 0.000) and social integration (β = 0.221, *p* = 0.000; β = 0.294, *p* = 0.000), while neither emotional support nor decisional support was significantly associated with elderly individuals’ self-rated physical health (β = 0.046, *p* > 0.05; β = 0.009, *p* > 0.05). Third, the results of the mediation analysis (Table 5) showed that self-rated physical health (β = 0.076, *p* = 0.000), self-rated mental state (β = 0.246, *p* = 0.000), and social integration (β = 0.199, *p* = 0.000) were significantly associated with elderly individuals’ living satisfaction. Therefore, this suggests that the mental state and social integration of the elderly individuals mediated the association between emotional support, decisional support, and living satisfaction, and no evidence of the mediating effect of self-rated physical health was observed. Therefore, full mediation occurred for the association between emotional support and living satisfaction because their association was no longer significant after controlling for the mediator. Partial mediation occurred for the association between decisional support and living satisfaction because the coefficient of the association decreased but still remained significant after controlling for the mediator.

**Figure 2:**
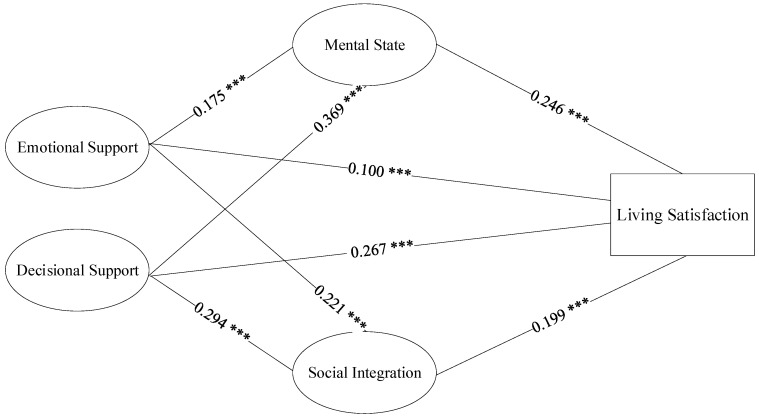
The mediating effect of multidimensional health on the relationship between family support and living satisfaction. Note: *** *p* < 0.001.

We also used the bootstrapping method to examine the mediating effect of the health variables and the results are presented in Table 6. First, the indirect effects of emotional support and decisional support on the living satisfaction of the elderly via mental state were significant (β = 0.043, *p* = 0.000, BCa 95% CI: (0.026, 0.063) β = 0.044, *p* = 0.000, BCa 95% CI: (0.029, 0.063)), and the mediator accounted for 43.05% of the total effect of emotional support on living satisfaction and 43.98% of the total effect of decisional support on living satisfaction. Second, the indirect effects of emotional support and decisional support on the living satisfaction of the elderly via social integration were significant (β = 0.091, *p* = 0.000, BCa 95% CI: (0.065, 0.121); β = 0.058, *p* = 0.000, BCa 95% CI: (0.038, 0.083)), and the mediator explained 34.00% of the total effect of emotional support on living satisfaction and 21.91% of the total effect of decisional support on living satisfaction. Finally, bias-corrected bootstrapped 95% confidence intervals for the path of emotional support and decisional support to the living satisfaction of the elderly via self-rated physical health included zero, indicating that a mediating effect of self-rated physical health did not exist, which is consistent with the results presented in Table 5. Figure 2 illustrates the results of the mediation analysis.

## 5. Discussion

This study investigated the relationship between family support and elderly individuals’ living satisfaction, considering different types of support. To further understand how different types of family support impact living satisfaction, we took three dimensions of health, namely, physical health, mental state, and social integration, as the moderators. To the best of our knowledge, this is the first study to test multidimensional health in a multiple mediation model in elderly people. Our findings revealed that emotional support and decisional support from families were positively related to the living satisfaction of elderly individuals, while the relationship between daily living support and living satisfaction was not significant. Mediation examination further demonstrated that both mental state and social integration mediated the association between emotional support and living satisfaction, as well as the association between decisional support and living satisfaction, but a mediating effect of physical health was not observed. Below, we elaborate more possible explanations for the results.

Hypothesis 1, which assumed that elderly individuals receiving daily living support from their families would be more satisfied with their life, was not supported. This finding is against our expectation but consistent with some studies conducted in Western countries and in East Asian contexts [50,51]. It is possible that the different types of support provided by families can reflect the demands of the elderly [52], and receiving daily living support and care may suggest functional dependencies and bad health conditions of older adults [20]. Simultaneously, studies have found that elderly people’s well-being may not increase when receiving support threatens autonomy, especially their autonomy and independence in their physical functioning [53]. Moreover, our findings can be explained by the theory of use and disuse. High-frequency daily living support may decrease the self-confidence and cognitive ability of the elderly and form path dependence on the support [54], thus leading to possible feelings of worthlessness and low satisfaction with life. Moreover, existing findings on filial piety in contemporary China have found that elderly parents have reconstructed their expectation of receiving daily living support from their offspring because of the increasing obstacles of geographical proximity and nuclearization of family. Consequently, the daily living support provided by children may not be regarded as much as a way of showing filial piety and thus may not affect older adults’ living satisfaction [55].

For emotional support, consistent with previous theoretical and empirical research results, our results demonstrated that emotional support from families was positively associated with the living satisfaction of elderly individuals [56,57], thus supporting hypothesis 2. The contribution of emotional support suggests that unlike daily living support, elderly individuals still hold high expectations of receiving emotional support from their families, especially support from their children and grandchildren, which plays an indispensable role in the conception of filial piety and is an important source of high elderly living satisfaction in China [58,59]. Furthermore, the socio-emotional selectivity theory posits that as people get older, they place more emphases on the emotional meaning of their life and optimize their social network to satisfy their emotional demands [60]. For Chinese elderly people, receiving emotional support from families is an essential component of their emotional demands [61]. With this support, elderly people seem to access the perceived availability and reliability of the family network, where they might feel supported and secure, which is a benefit to their personal well-being [8].

For decisional support, in accordance with hypothesis 3, elderly individuals who receive decisional support from families had higher living satisfaction than those who did not receive any decisional support. There is substantial evidence that social support provided by important roles in an individual’s life shapes their sense of personal worth and self-esteem [62]. Lou (2010) believed that support from grandchildren brings a sense of self-control and self-esteem to grandparents in Hong Kong [58]. As the core part of Chinese elderly people’s social networks, families and their support are crucial in increasing elderly individuals’ self-esteem, which is integral to their well-being and living satisfaction.

In terms of the mediation effect, as per the requirement of the mediating models recommended by Baron and Kenny (1986), since the association between daily living support and elderly living satisfaction was not significant, hypothesis 4, hypothesis 5, and hypothesis 6 were also invalid in this study. Furthermore, for physical health, hypothesis 7 and hypothesis 10, which assumed that physical health mediates the association between emotional support and living satisfaction, as well as the association between decisional support and the living satisfaction of elderly individuals, were not supported. According to the disengagement theory, aging is irreversible, and in this process, the decline of physical fitness, the brain, and cognition is inevitable [63]. Meanwhile receiving emotional support and decisional support from families instead of professional medical care may not slow down elderly individuals’ inescapable physical and cognitive decline [20], which is in accordance with the results of the regression and mediation analyses in this study (Table 5). Therefore, this might help to explain the absence of a mediating effect of physical health on the relationship between emotional support and living satisfaction, as well as the association between decisional support and living satisfaction in this study.

As expected, this study found that mental state mediates the relationship between emotional support and living satisfaction, as well as the association between decisional support and living satisfaction of the Chinese elderly, supporting hypothesis 8 and 11. First, the results are consistent with previous studies regarding the positive association between emotional support and mental health [40], as well as the positive association between mental health and living satisfaction of the elderly [27,28,29,30]. This study provided further evidence that emotional support from families has a positive impact on the living satisfaction of the elderly via affecting their mental state. This finding is in line with the viewpoint of Wang et al. (2020), which proposed that an indirect effect of social support exists through depression in relation to quality of life among rural older Chinese [64]. As family members place in the innermost circle of elderly people’s social networks, the companionship and concern provided by them can meet the essential emotional demands of the elderly, which alleviates their loneliness and mental stress, and consequently increases their living satisfaction. Second, our results on the mediating role of mental state in the association between decisional support and living satisfaction expand the findings from previous studies, which proved that higher self-esteem was useful for dealing with psychological distress and thus promotes one’s quality of life [65,66]. Receiving decisional support from families can make the elderly feel involved in family activities and respected by families and can increase elderly individuals’ feelings of self-acceptance and self-esteem, which is beneficial to reduce their risk of depression and other mental problems, and further contributes to their living satisfaction.

In addition, the mediating effects of social integration on the association between emotional support and living satisfaction, as well as the association between decisional support and living satisfaction, were also verified in this study, supporting hypothesis 9 and hypothesis 12. Accumulating evidence indicates that family support correlates with elderly people’s social integration, as measured by social participation or social engagement [67,68], and social integration has an important role in maintaining well-being in old age [35,69]. Expanding prior research, this study employed three indexes, including social participation, social relationships, and social identity, to measure social integration and further verified the underlying mechanism by which emotional support and decisional support from families indirectly affect the living satisfaction of elderly individuals by increasing their social integration level. Echoing these findings, this study suggested that receiving emotional support and decisional support from families can increase elderly people’s sense of security and self-confidence, which is beneficial to promote their aspirations to take part in social activities and to enhance their social integration. Consequently, in line with the active aging theory [70], an increase of social integration level is conductive to boosting the living satisfaction of the elderly.

This study has several important practical implications for enhancing the living satisfaction of Chinese elderly people. The key findings of this study suggest that it is the emotional and decisional dimensions of family support that are effective in promoting the living satisfaction of elderly individuals, whereas the positive effect of daily living support is not significant. Moreover, our results revealed that the contribution of decisional support provided by families to the elderly individuals’ living satisfaction is higher compared to emotional support. As such, there is a need to pay extra attention to elderly individuals’ demands for family support, particularly in terms of emotional support and decisional support. In fact, with the rapid socioeconomic change in China, Chinese legal systems and a series of national policy plans have been developed to stress and promote the essential role of the family in supporting older adults [71]. Nevertheless, many family members, especially adult children, only provide some instrumental and financial support to older adults and overlook their needs for company and respect. Therefore, to effectively improve the living satisfaction and holistic well-being of Chinese elderly people, the government should realize the change in elderly individuals’ demands for family support, and targeted social policies should be made to encourage and guide the family to concern and satisfy the emotional care and esteem demands of their older family members.

Furthermore, this study highlights the importance of mental state and social integration as complementary mechanisms that contribute to explaining the indirect effect of emotional support and decisional support on the living satisfaction of Chinese elderly people. This finding may help policy-makers to better understand the mechanisms of how emotional support and decisional support affect the living satisfaction of elderly individuals and the important effect of mental state and social integration on the living satisfaction of older adults, thus designing and implementing more targeted policy measures to promote the mental health and social well-being of older adults. For instance, the government should speed up the establishment of a diversified and multilevel old age service system in China, including community care services, to supplement the deficiency of family support in promoting older adults’ mental health and social well-being, and to further enhance their living satisfaction.

There are some limitations of this study. First, the main limitation of this study is that the relationships among family support, multidimensional health, and living satisfaction of the elderly were examined using a cross-sectional method, which may have ignored the possible time lag between family support and its outcome variables, whereas longitudinal studies may provide further validation. Second, due to the restriction of the study design, we were not able to measure aspects such as level of cognitive impairment and other potential confounding factors that could affect the living satisfaction of the elderly, and so the results may be biased. Third, the survey was conducted only in Shaanxi province, an important western province representing an intermediate economy level in China, and the results may not be applicable to other provinces with different socioeconomic statuses. Therefore, more provinces should be included in future studies to explore the differences among regions.

## 6. Conclusions

Our study demonstrated the significant associations between different types of family support and the living satisfaction of Chinese elderly people, and further revealed that the associations were mediated by the effects of mental state and social integration. This study emphasized the effect of emotional support and decisional support on promoting the mental health and social well-being of the elderly and on further boosting their living satisfaction. These results have significant implications for enhancing the living satisfaction and holistic well-being of Chinese elderly people.

## Figures and Tables

**Table 1:** Definition/codes of the sociodemographic variables.

Variable	Code/Definition
Age	Continuous variable
Gender	0 = Male; 1 = Female
Marital status	0 = Non-single; 1 = Single (contains unmarried, divorced, and widowed)
Household income	Categorical variable (<¥10,000; ¥10,000–¥50,000; ¥50,000–¥100,000; ¥100,000–¥200,000; >¥200,000)
Hukou location	0 = Urban; 1 = Rural
Education level	Categorical variable (primary school or lower; junior middle school; senior middle school or higher)
Living mode	0 = Living in the home/community; 1 = Living in an old age care institution
Having any ADL limitation	0 = Yes; 1 = No
Having any chronic disease	0 = Yes; 1 = No
Health insurance	0 = Yes; 1 = No
Old age insurance	0 = Yes; 1 = No

Note: ADL, Activities of Daily Living.

**Table 2:** Descriptive statistics of the sociodemographic variables (*N* = 938).

Variable		Frequency (*n*)/Mean	Percentage (%)/SD
Age	(Mean, SD)	72.19	8.36
Gender	Male	402	42.86
	Female	536	57.14
Marital status	Non-single	406	43.28
	Single	532	56.72
Household income	<¥10,000	132	14.07
	¥10,000–¥50,000	200	21.32
	¥50,000–¥100,000	99	10.55
	¥100,000–¥200,000	319	34.01
	>¥200,000	188	20.04
Hukou location	Urban	514	54.80
	Rural	424	45.20
Education level	Primary school or lower	447	47.65
	Junior middle school	247	26.33
	Senior middle school or higher	244	26.01
Living mode	Living in the home/community	687	73.24
	Living in an old age care institution	251	26.76
Having any ADL limitation	Yes	76	8.10
Having any chronic disease	Yes	529	56.40
Health insurance	Yes	914	97.44
Old-age insurance	Yes	887	94.56

Note: SD, standard deviation.

**Table 3:** Living satisfaction, family support, and heath characteristics of the participants.

Variable		Minimum	Maximum	Mean	SD
Before data normalization	Living satisfaction	7	25	19.061	3.053
	Family support				
	Daily living support	1	5	3.472	1.462
	Emotional support	1	5	4.095	1.096
	Decisional support	1	5	4.259	0.731
	Multidimensional health				
	Self-rated physical health	1	5	3.383	1.095
	Mental state	1	5	4.168	0.939
	Social integration	3	15	12.978	2.029
After data normalization	Living satisfaction	0	1	0.670	0.169
	Family support				
	Daily living support	0	1	0.618	0.365
	Emotional support	0	1	0.774	0.274
	Decisional support	0	1	0.815	0.183
	Multidimensional health				
	Self-rated physical health	0	1	0.596	0.274
	Mental state	0	1	0.792	0.235
	Social integration	0	1	0.817	0.184

Note: SD, standard deviation. Data normalization: (X − Min Value)/(Max Value − Min Value). The results of the data normalization of the control variables are not present due to the space constraints.

**Table 4:** Multivariate linear regression models of living satisfaction.

Variable	Model 1 β (SE)	Model 2 β (SE)	Model 3 β (SE)
Age	−0.011 (0.024)	−0.022 (0.022)	0.005 (0.019)
Gender	0.003 (0.010)	−0.005 (0.009)	0.003 (0.008)
Marital status	0.021 (0.012)	0.006 (0.011)	0.004 (0.009)
Household income			
¥10,000–¥50,000	0.067 (0.016) ***	0.044 (0.015) **	0.023 (0.013)
¥50,000–¥100,000	0.116 (0.020) ***	0.087 (0.018) ***	0.056 (0.016) ***
¥100,000–¥200,000	0.160 (0.015) ***	0.124 (0.015) ***	0.080 (0.013) ***
>¥200,000	0.228 (0.017) ***	0.174 (0.017) ***	0.125 (0.015) ***
Hukou location	0.013 (0.011)	0.028 (0.010) **	0.021 (0.008) *
Education level			
Junior middle school	0.033 (0.013) *	0.026 (0.012) *	0.020 (0.010)
Senior middle school or higher	0.034 (0.013) *	0.025 (0.012) *	0.023 (0.010) *
Living mode	0.046 (0.014) **	0.060 (0.013) ***	0.045 (0.011) ***
Having any ADL limitation	0.109 (0.019) ***	0.080 (0.017) ***	0.018 (0.015)
Having any chronic disease	0.045 (0.010) ***	0.037 (0.009) ***	0.012 (0.008)
Health insurance	0.035 (0.022)	0.032 (0.029)	0.029 (0.024)
Old age insurance	0.034 (0.045)	0.018 (0.020)	0.013 (0.017)
Family support			
Daily living support		0.017 (0.014)	0.012 (0.012)
Emotional support		0.101 (0.019) ***	0.010 (0.016)
Decisional support		0.263 (0.027) ***	0.114 (0.024) ***
Multidimensional health			
Self-rated physical health			0.073 (0.016) ***
Mental state			0.246 (0.021) ***
Social integration			0.199 (0.026) ***
Observation	938	938	938
Adjusted R-square	0.264	0.390	0.569

Note: β, regression coefficient; SE, standard error. * *p* < 0.05, ** *p* < 0.01, and *** *p* < 0.001.

**Table 5:** The results of the regression and mediation analyses.

Variable	Model 1 β (SE)	Model 2 β (SE)	Model 3 β (SE)	Model 4 β (SE)	Model 5 β (SE)
	Y: Living Satisfaction	M1: Self-Rated Physical Health	M2: Mental State	M3: Social Integration	Y: Living Satisfaction
Emotional support	0.100 (0.019) ***	0.046 (0.034)	0.175 (0.027) ***	0.221 (0.022) ***	0.010 (0.016)
Decisional support	0.267 (0.027) ***	0.009 (0.049)	0.369 (0.039) ***	0.294 (0.032) ***	0.118 (0.024) ***
Self-rated physical health					0.076 (0.015) ***
Mental state					0.246 (0.021) ***
Social integration					0.199 (0.026) ***
CV	Yes	Yes	Yes	Yes	Yes
Observation	938	938	938	938	938
Adjusted R-squared	0.389	0.218	0.305	0.276	0.569

Note: β, regression coefficient; SE, standard error; CV, control variable. * *p* < 0.05, ** *p* < 0.01, and *** *p* < 0.001.

**Table 6:** The results of the bootstrap analysis test.

Mediator	Emotional Support–Health–Living Satisfaction			Decisional Support–Health–Living Satisfaction		
	Indirect Effect	BCa 95% CI		Indirect Effect	BCa 95% CI	
	β (SE)	Lower	Upper	β (SE)	Lower	Upper
Multidimensional health						
Self-rated physical health	0.003 (0.003)	−0.001	0.011	0.001 (0.004)	−0.007	0.009
Mental state	0.043 (0.009) ***	0.026	0.063	0.091 (0.014) ***	0.065	0.121
Social integration	0.044 (0.009) ***	0.029	0.063	0.058 (0.011) ***	0.038	0.083

Note: Based on 5000 bootstrap samples. Β, bootstrap estimate coefficient; SE, standard error. BCa, bias-corrected and accelerated; CI, confidence interval. * *p* < 0.05, ** *p* < 0.01, and *** *p* < 0.001.
